# Capsicum Cough

**Published:** 1887-10

**Authors:** E. W. Berridge


### CAPSICUM COUGH.

E. W. Berridge, M. D.

1886, November 10th, Miss S. H. complained of cough, causing drawing pain from lower chest in middle line up to throat. Capsicum $^{20m}$ (F. C.) one dose.

She was quickly relieved; the drawing pain went first, then the cough.

